# Only the new beginning of VAP quality control

**DOI:** 10.1186/s13054-023-04358-0

**Published:** 2023-03-13

**Authors:** Xin Ding, Xudong Ma, Xiang Zhou

**Affiliations:** 1 Department of Critical Care Medicine, State Key Laboratory of Complex Severe and Rare Diseases, Peking Union Medical College Hospital, Peking Union Medical College and Chinese Academy of Medical Sciences, Beijing, 100730 China; 2 Department of Medical Administration, National Health Commission of the People’s Republic of China, Beijing, 100044 China; 3 Information Center Department, State Key Laboratory of Complex Severe and Rare Diseases, Peking Union Medical College Hospital, Peking Union Medical College & Chinese Academy of Medical Sciences, Beijing, 100730 China

Dear editor:

We would like to thank to Dr Wang and colleagues for their interest and comments about our article [1]. Before we respond to their comments, we have to explain that the data from the National Clinical Improvement System were uploaded by hospitals and were the descriptive data of the hospital profile rather than individual cases, which was mentioned as one of the limitations in our article [2]. Therefore, the information of other factors such as ventilator days, age, gender, and comorbidities of every case were not included in the database. As the number of cases diagnosed and died of VAP among different hospitals were counting data, which were approximately following Poisson distribution, Poisson regression model was better to answer the questions such as what factors can influence the VAP incidence rate and mortality in this case.

Regarding the first question about the ventilator days, it is the fact that patient transfer between hospitals is very common. As the VAP incidence rate is used as one of the ICU quality control indicators, it is defined as the number of cases diagnosing VAP divided by the ventilator days during the same period in the ICU. The ventilator days of the patients transferred from other hospital with endotracheal intubation before admission were calculated in the previous hospitals.

The authors mentioned the heterogeneity of different specialized ICU and different hospital levels. First, as the data were collected by the China-National Critical Care Quality Control Center (China-NCCQC), although the ICUs in specialized hospitals such as Gynecologic and Obstetric hospital were included, specialized ICUs such as Respiratory ICU in general hospital were not included. As the doctors in specialized ICU received less training on nosocomial infection and were allocated with less resource, VAP incidence rate and mortality may be worse in these ICUs [3]. Although it is generally believed that tertiary hospitals are usually more adequately staffed and equipped compared to secondary hospitals, which may lead to a lower rate of nosocomial infections, it is not the case at least for VAP [4]. Considering the heterogeneity of development between different regions, the level of hospital may not be the unique standard to estimate the quality. For example, the ICUs in secondary hospitals from high GDP level area may be better than these in tertiary hospitals from low GDP level area.

We are glad to find that our research can raise the attention on VAP in ICU specialists, and this study was only the beginning of the VAP quality control. New research based on individual cases is on the way, and we hope the results could bring us more satisfactory answers to all those questions.

## Data Availability

Not applicable.
